# An Interactive Association of Advanced Glycation End-Product Receptor Gene Four Common Polymorphisms with Coronary Artery Disease in Northeastern Han Chinese

**DOI:** 10.1371/journal.pone.0076966

**Published:** 2013-10-14

**Authors:** Xiaohong Yu, Jun Liu, Hao Zhu, Yunlong Xia, Lianjun Gao, Zhen Li, Nan Jia, Weifeng Shen, Yanzong Yang, Wenquan Niu

**Affiliations:** 1 Department of Cardiology, The First Affiliated Hospital of Dalian Medical University, Dalian, Liaoning, China; 2 Department of Hypertension, Ruijin Hospital, School of Medicine, Shanghai Jiao Tong University, Shanghai, China; 3 Department of Cardiology, Ruijin Hospital, School of Medicine, Shanghai Jiao Tong University, Shanghai, China; 4 State Key Laboratory of Medical Genomics, Ruijin Hospital, School of Medicine, Shanghai Jiao Tong University, Shanghai, China; 5 Shanghai Institute of Hypertension, School of Medicine, Shanghai Jiao Tong University, Shanghai, China; University of Miami, United States of America

## Abstract

**Background:**

Growing evidence indicates that advanced glycation end-product receptor (RAGE) might play a contributory role in the pathogenesis of coronary artery disease (CAD). To shed some light from a genetic perspective, we sought to investigate the interactive association of *RAGE* gene four common polymorphisms (rs1800625 or T-429C, rs1800624 or T-374A, rs2070600 or Gly82Ser, and rs184003 or G1704A) with the risk of developing CAD in a large northeastern Han Chinese population.

**Methodology/Principal Findings:**

This was a hospital-based case-control study incorporating 1142 patients diagnosed with CAD and 1106 age- and gender-matched controls. All individuals were angiographically confirmed. Risk estimates were expressed as odds ratio (OR) and 95% confidence interval (CI). Overall there were significant differences in the genotype and allele distributions of rs1800625 and rs184003, even after the Bonferroni correction. Logistic regression analyses indicated that rs1800625 and rs184003 were associated with significant risk of CAD under both additive (OR = 1.20 and 1.23; 95% CI: 1.06–1.37 and 1.06–1.42; P = 0.006 and 0.008) and recessive (OR = 1.75 and 2.39; 95% CI: 1.28–2.40 and 1.47–3.87; P<0.001 and <0.001) models after adjusting for confounders. In haplotype analyses, haplotypes C-T-G-G and T-A-G-T (alleles in order of rs1800625, rs1800624, rs2070600 and rs184003), overrepresented in patients, were associated with 52% (95% CI: 1.19–1.87; P = 0.0052) and 63% (95% CI: 1.14–2.34; P = 0.0075) significant increases in adjusted risk for CAD. Further interactive analyses identified an overall best multifactor dimensionality reduction (MDR) model including rs1800625 and rs184003. This model had a maximal testing accuracy of 0.6856 and a cross-validation consistency of 10 out of 10 (P = 0.0016). The validity of this model was substantiated by classical Logistic regression analysis.

**Conclusions:**

Our findings provided strong evidence for the potentially contributory roles of *RAGE* multiple genetic polymorphisms, especially in the context of locus-to-locus interaction, in the pathogenesis of CAD among northeastern Han Chinese.

## Introduction

Advanced glycation end-product receptor (protein: RAGE; gene: *RAGE*) is a member of the immunoglobulin superfamily of cell surface receptors, and it interacts with advanced glycation end-products and other molecules implicated in inflammation, atherogenesis and vasoconstriction, eventually leading to coronary dysfunction, atherosclerosis and thrombosis [1]–[3]. Evidence is mounting from animal experiments suggesting the protection against inflammatory conditions, heart failure, and coronary artery disease (CAD) after the pharmacological blockade of RAGE or the genetic knockout of *RAGE* [4], [5]. In humans, soluble forms of RAGE or sRAGE in plasma can predict the development and progression of heart failure, irrespective of the presence of diabetes [6]. Likewise, plasma sRAGE levels were negatively associated with the angiographically-confirmed CAD, and this association was dose-dependent with patients in the lowest quartile of sRAGE exhibiting the highest risk of CAD [7]. On the basis of these observations, it is reasonable to speculate that *RAGE* gene might play a contributory role in the pathogenesis of CAD.

The gene encoding *RAGE* is highly polymorphic, and more than twenty polymorphisms so far have been validated. Best evaluated with respect to the association with CAD or related intermediate phenotypes in *RAGE* gene are four common polymorphisms, *viz*. rs1800625 (T-429C) and rs1800624 (T-374A) in the promoter region, rs2070600 (Gly82Ser) in 3^rd^ exon, and rs184003 (G1704A) in 7^th^ intron. Despite a large panel of the *RAGE*-CAD genetic association studies, it remains unclear whether individuals possessing the genetic defect (s) of these polymorphisms, in isolation or in combination, are more susceptible to CAD than those with the alternative one (s) [8]–[11]. To make definitive claims about the involvement of *RAGE* gene in the development of CAD, comprehensive genetic approaches such as replication studies with other populations have attracted special attention. To generate more information, we sought to investigate the interactive association of these four common polymorphisms in *RAGE* gene with the risk of developing CAD in a large northeastern Han Chinese population.

## Methods

### Study population

This study was conducted on a hospital-based case-control design involving 2248 unrelated individuals admitted to the Department of Cardiology, the First Affiliated Hospital of Dalian Medical University. All study individuals were Han Chinese and resided in Dalian city, Liaoning province, and they were classified into CAD group and control group according to the angiographic results. Coronary angiography was undertaken by the standard Judkins techniques or through the radial approach. The CAD group enrolled was angiographically confirmed in the presence of more than 50% stenosis in at least one of the three major coronary arteries or major branches. Patients were excluded if they had simple spasm of coronary arteries, myocardial bridge or other non-coronary atherosclerotic lesions. The controls had no history of any vascular event and had normal coronary arteries on angiography. In total, there were 1142 patients diagnosed with CAD and 1106 age- and gender-matched controls.

All individuals signed written informed consent prior to enrollment. This study was reviewed and approved by the Ethics Committee of Dalian Medical University, and was conducted in agreement with the Declaration of Helsinki Principles.

### Study characteristics

At enrollment, body weight and height were recorded, and body mass index (BMI) was calculated as weight in kilograms divided by height in meters squared. Systolic and diastolic blood pressures (SBP and DBP) at sitting position were measured twice with a five-minute interval by certified nurses.

Venous blood was extracted from each individual after an overnight fasting of at least 8 hours. Fasting glucose was measured in fluoride plasma by an electrochemical glucose oxidase method. Plasma levels of triglyceride (TG), total cholesterol (TC), high-density lipoprotein cholesterol (HDL-C), lipoprotein (a), blood urea nitrogen (BUN), creatinine and urea acid (UA) were determined enzymatically using available kits and auto analyzers. Plasma high sensitivity C-reactive protein (hsCRP) levels were determined using the high-sensitivity enzyme-linked immunosorbent assay (ELISA) kit.

### Genotyping

Genomic DNA was obtained from peripheral blood leukocytes by TIANamp Blood DNA Kit (Tiangen Biotect (Beijing) Co., China) and was stored at −40°C until required for batch genotyping. Plasma was prepared for quantifying routine biological profiles.

All polymorphisms were genotyped according to the polymerase chain reaction-ligase detection reaction (PCR-LDR) method as previously described [12]. The primers for PCR amplification and the probes for LDR can be obtained by request. PCR reactions were performed in the EDC-810 Amplifier.

For each polymorphism, two specific probes were synthesized to discriminate specific bases, and additionally one common probe was synthesized and labeled at the 3′ end with 6-carboxy-fluorescein (FAM) and phosphorylated at the 5′ end. The multiplex ligation reaction was carried out in a reaction volume of 10 μl containing 2 μl of PCR product, 1 μl 10×Taq DNA ligase buffer, 1 μM of each discriminating probe, 5 U Taq DNA ligase, and the ligation parameters were 30 cycles of 94°C for 30 seconds and 56°C for 3 minutes. After reaction, 1 μl LDR reaction product was mixed with 1 μl ROX passive reference and 1 μl loading buffer, and then denatured at 95°C for 3 minutes, chilled rapidly in ice water. The fluorescent products of LDR were differentiated using ABI 3730XL sequencer (Applied Biosystems, USA).

### Statistical analysis

Pearson χ^2^ and unpaired Student's t-test or Mann-Whitney U test were adopted to examine the differences between CAD patients and controls for categorical (including genotypes and alleles of examined polymorphisms) and continuous variables, respectively. Testing for deviations from Hardy-Weinberg equilibrium was carried out using a Pearson goodness-of-fit test. Two-tailed P<0.05 was accepted as statistical significance.

Each genotype of examined polymorphisms was assessed by Logistic regression analyses under the additive (major homozygotes versus heterozygotes versus minor homozygotes), dominant (major homozygotes versus heterozygotes plus minor homozygotes) and recessive (major homozygotes plus heterozygotes versus minor homozygotes) models of inheritance after adjusting for confounding factors, respectively.

The haplotype frequencies of four examined polymorphisms in *RAGE* gene were estimated by haplo.em program, which computes the maximum likelihood estimates of haplotype probabilities using the progressive insertion algorithm which progressively inserts batches of loci into haplotypes of growing lengths. Only haplotype with frequency ≥3% was considered in haplotype analyses. The haplo.cc and haplo.glm programs were employed to calculate the adjusted odds ratios (ORs) and 95% confidence intervals (CIs) for each haplotype. These two programs are based on a generalized linear model, and compute the regression of a trait on haplotypes and other covariates [13]. Simulated P values were calculated based on 1000 replicates. All mentioned haplo.* programs were implemented in Haplo.Stats software (version 1.4.0) operated in the R language (version 2.14, available at the website http://www.r-project.org).

Interactive analyses were conducted in the open-source multifactor dimensionality reduction (MDR) software (version 2.0) (www.epistasis.org) [14], [15]. All possible combinations of four examined polymorphisms were constructed using MDR constructive induction. The accuracy of each model was evaluated by a Bayes classifier in the context of 10-fold cross-validation. In general, a single best model simultaneously has the maximal testing accuracy and cross-validation consistency. The cross-validation consistency is a measure of the number of times of 10 divisions of the dataset that the best model is extracted. Statistical significance was evaluated using a 1000-fold permutation test to compare observed testing accuracies with those expected under the null hypothesis of null association. Permutation testing corrects for multiple testing by repeating the entire analysis on 1000 datasets that are consistent with the null hypothesis. Further to validate the soundness of MDR method, a classical Logistic regression analysis was undertaken to check the derived best model.

Statistical analyses were conducted by STATA software v11.0 for Windows (StataCorp LP, College Station, TX, USA). Study power was estimated by adopting the Power and Sample Size Calculations (PS) software (v3.0.7) [6]. The linkage disequilibrium was performed by Haploview (v.4.0), and the linkage disequilibrium coefficient was expressed as D'.

## Results

### Baseline characteristics

Differences of study characteristics between CAD group and control group are compared in Table 1. Age and gender distributed similarly between the two groups. CAD patients had relatively higher BMI than controls (P = 0.0637). Blood pressures and fasting glucose levels were strikingly higher in patients than in controls (P<0.0005). Plasma total cholesterol and HDL-C levels were significantly lower in patients than in controls (P<0.0005). In contrast, plasma lipoprotein (a) (P<0.0005), creatinine (P = 0.0006) and hsCRP (P<0.0005) levels were significantly higher in patients than in controls. There were no significant differences for BUN and uric acid.

**Table 1:** Baseline characteristics of study population.

Characteristics	CAD patients (n = 1142)	Controls (n = 1106)	P
Age, years	62.07±9.07	62.42±9.85	0.3749
Gender (Males)	46.58%	49.10%	0.2334
BMI, kg/m^2^	26.19±15.32	24.9±3.64	0.0637
SBP, mmHg	141.44±16.82	137.31±20.52	<0.0005
DBP, mmHg	84.86±10.63	81.09±11.92	<0.0005
Fasting glucose, mmol/L	6.14±2.15	5.47±1.26	<0.0005
Triglycerides, mmol/L	1.9±1.04	1.92±1.45	0.7240
Total cholesterol, mmol/L	4.59±1.18	4.81±1.0	<0.0005
HDL-C, mmol/L	1.12±0.32	1.35±0.4	<0.0005
LDL-C, mmol/L	2.75±0.95	2.75±0.77	0.8986
Lipoprotein (a), mmol/L	0.3±0.45	0.21±0.19	<0.0005
BUN, mmol/L	5.92±3.89	5.76±3.71	0.3794
Creatinine, μmol/L	87.49±36.81	81.35±35.96	0.0006
Uric acid, μmol/L	329.06±100.37	328.85±92.52	0.9644
hsCRP, mmol/L	12.37±11.42	2.21±3.71	<0.0005

*Abbreviations:* CAD, coronary artery disease; BMI, body mass index; HDL-C, high-density lipoprotein cholesterol; LDL-C, low-density lipoprotein cholesterol; BUN, blood urea nitrogen; hsCRP, high sensitivity C-reactive protein. Data are expressed as mean ± standard deviation unless otherwise indicated.

### Single-locus analyses

The genotype distributions and allele frequencies of four examined polymorphisms in *RAGE* gene and their risk prediction for CAD are summarized in Table 2. There was no detectable deviation from the Hardy-Weinberg equilibrium for all polymorphisms in both patients and controls (P>0.05). Overall there were statistically significant differences in the genotypes and alleles of rs1800625 and rs184003, even after applying a Bonferroni correction to account for multiple testing with respect to the four polymorphisms (P<0.0125). Correspondingly, the power to reject the null hypothesis of no difference in genotype frequencies for rs1800625 and rs184003 between patients and controls was 94.4% and 99.6%, respectively. No significance was reached for the other two polymorphisms under study. Moreover, considering the absolute linkage disequilibrium between rs1800625 and rs1800624 reported in Euro- and Afro-Brazilians [16], the relation of these two polymorphisms was checked in all individuals, and the linkage disequilibrium was only moderate (D' = 0.67), indicating the potential existence of genetic heterogeneity across ethnicities.

**Table 2:** Genotype distributions and allele frequencies of four examined polymorphisms in *RAGE* gene between patients and healthy controls and their risk prediction for coronary artery disease.

Polymorphisms		CAD patients (n = 1142)	Controls (n = 1106)	P_χ2_	OR; 95% CI; P^*^
**rs1800625**	TT	557	577		1.20; 1.06–1.37; 0.0061.14; 0.97–1.35; 0.1141.75; 1.28–2.40; <0.001
Genotype (n):	TC	468	461	0.002	
	CC	117	68		
Allele (%):	C	30.74	26.99	0.006	
**rs1800624**	TT	608	604		1.04; 0.92–1.19; 0.5151.06; 0.9–1.25; 0.5011.05; 0.78–1.41; 0.759
Genotype (n):	TA	436	410	0.808	
	AA	98	92		
Allele (%):	A	27.67	26.85	0.538	
**rs2070600**	GG	482	496		1.11; 0.98–1.26; 0.0861.11; 0.94–1.31; 0.2351.26; 0.97–1.62; 0.079
Genotype (n):	GA	507	489	0.158	
	AA	153	121		
Allele (%):	A	35.6	33.05	0.072	
**rs184003**	GG	729	742		1.23; 1.06–1.42; 0.0081.16; 0.98–1.38; 0.0912.39; 1.47–3.87; <0.001
Genotype (n):	GT	355	339	0.001	
	TT	58	25		
Allele (%):	T	20.62	17.59	0.011	

*Abbreviations:* CAD, coronary artery disease; OR, odds ratio; 95% CI, 95% confidence interval. * OR, 95% CI, and P values were calculated under the additive (the upper), dominant (the middle), and recessive (the lower) models of inheritance after adjusting for age, gender, body mass index, systolic blood pressure, and fasting glucose.

Three models of inheritance including additive, dominant and recessive models were explored for each polymorphism. Results from Logistic regression analyses indicated that rs1800625 and rs184003 were significantly associated with the risk of having CAD under both additive (OR = 1.20 and 1.23; 95% CI: 1.06–1.37 and 1.06–1.42; P = 0.006 and 0.008, respectively) and recessive (OR = 1.75 and 2.39; 95% CI: 1.28–2.40 and 1.47–3.87; P<0.001 and <0.001, respectively) models after adjusting for age, gender, BMI, SBP and fasting glucose.

### Haplotype analyses

Table 3 presents the haplotype frequencies (≥3%) of four examined polymorphisms in patients and controls with the cumulative frequencies reaching 92.89% and 88.83% respectively. The most common haplotype T-T-A-G (alleles in order of rs1800625, rs1800624, rs2070600 and rs184003) was comparable in frequencies between patients and controls (P_Sim_ = 0.1026), and was assigned as the reference group in risk estimates. Haplotypes C-T-G-G and T-A-G-T, which were remarkably overrepresented in patients, were respectively associated with a 52% (95% CI: 1.19–1.87; P = 0.0052) and 63% (95% CI: 1.14–2.34; P = 0.0075) increased risk of developing CAD after adjusting for age, gender, BMI, SBP and fasting glucose. Accordingly for these two haplotypes, the power to reject the null hypothesis of no difference between patients and controls was 98.6% and 99.1%, respectively.

**Table 3:** Haplotype frequencies of four polymorphisms examined in *RAGE* gene between patients and controls and their risk prediction for coronary artery disease.

Haplotype^*^	CAD patients	Controls	P_Sim_	OR; 95% CI; P^†^
T-T-G-G	25.68	26.36	0.1026	Reference group
T-T-A-G	16.44	17.94	0.1225	0.97; 0.76–1.21; 0.6274
C-T-G-G	13.95	9.92	0.0038	1.52; 1.19–1.87; 0.0052
T-A-G-G	9.08	11.9	0.009	0.79; 0.52–1.06; 0.1115
C-T-A-G	7.72	6.17	0.0387	1.33; 0.91–1.82; 0.1397
T-T-G-T	6.64	4.41	0.0184	1.53; 0.97–2.11; 0.0806
T-A-G-T	5.75	3.1	0.0091	1.63; 1.14–2.34; 0.0075
T-A-A-G	4.75	5.48	0.2145	0.98; 0.66–1.46; 0.8347
C-A-G-G	2.88	3.55	0.2798	0.91; 0.53–1.37; 0.7904

*Abbreviations:* CAD, coronary artery disease, P_Sim_, simulated P value; OR, odds ratio; 95% CI, 95% confidence interval. P_Sim_ was calculated based on randomly permuting the trait and covariates and then computing the haplotype score statistics. *Alleles in haplotype were presented in order of polymorphisms rs1800625, rs1800624, rs2070600 and rs184003. **^†^**OR, 95% CI, and P values were calculated after considering age, gender, body mass index, systolic blood pressure, and fasting glucose as covariates.

### Interactive analyses

To shed some light on the potential genetic interactions, an exhaustive MDR analysis that evaluates all possible combinations of four examined polymorphisms in *RAGE* gene is shown in Table 4. Specifically, each best model was accompanied with the testing accuracy, cross-validation consistency and significant level as determined by permutation testing. The overall best MDR model included rs1800625 and rs184003, and this model had a maximal testing accuracy of 0.6856 and a cross-validation consistency of 10 out of 10. Moreover, this model was significant at the level of 0.0016, indicating that a model this good or better was observed only by less than 2 out of 1000 permutations and was thus unlikely under the null hypothesis of null association.

**Table 4:** MDR analysis summary.

Best combination of each model	Cross-validation consistency	Testing accuracy	P
rs1800625	8	0.6243	0.0637
rs1800625, rs184003	10	0.6856	0.0016*
rs1800625, rs1800624, rs184003	9	0.6637	0.0039
rs1800625, rs1800624, rs2070600, rs184003	10	0.6709	0.0021

* The overall best MDR model.

To further validate the predictive value of MDR model, classical Logistic regression analysis was employed accordingly. The interaction of rs1800625 and rs184003 (rs1800625*rs184003) was associated with 1.12-fold (95% CI: 1.05–1.2; P = 0.001), 1.09-fold (95% CI: 1.01–1.9; P = 0.031) and 1.83-fold (95% CI: 1.42–2.36; P<0.0005) increased risk of having CAD under additive, dominant and recessive models of inheritance after adjusting for age, gender, BMI, SBP and fasting glucose.

## Discussion

In the present study, we sought to investigate the association of *RAGE* gene four common polymorphisms with the risk of developing CAD in a large northeastern Han Chinese population involving 2248 individuals. The principal finding was the potential interactive roles of *RAGE* gene rs1800625 (T-429C) and rs184003 (G1704A) in the development of CAD. To the best of our knowledge, this report so far is the largest case-control association study examining the susceptibility of *RAGE* multiple genetic polymorphisms to CAD in Chinese.

More recently, Wang and colleagues have conducted a meta-analysis by synthesizing data from 17 studies on *RAGE* gene three polymorphisms (T-429C, T-374A, Gly82Ser) and the risk of CAD, but unfortunately they failed to detect any suggestive association [17]. This negative finding is possibly due to genetic heterogeneity that is not uncommon in any disease identification strategy [18], where this heterogeneity can be somewhat avoided when homogeneous populations are used [19]. Factually in this study, all study individuals are of Han descent and local residents of northeastern regions of China. They are characterized by genetic homogeneity and geographic stability, and are probably more uniform in their environmental exposures, including the habitual dietary intake of high salt and high fat. All these characteristics render this population more appropriate to enhance our understanding of genetic architecture of CAD and related intermediate phenotypes such as blood pressure. Moreover, it cannot be totally ruled out that the evolutionary history of linkage disequilibrium patterns will vary significantly in different ethnic populations. For example, the degrees of linkage disequilibrium between rs1800625 and rs1800624 were differentiated between Euro- and Afro-Brazilians [16] and Han Chinese in this study. Further in this study all examined polymorphisms respected the Hardy-Weinberg equilibrium in both patients and controls, lowering the likelihood of being biased by faulty genotyping or population stratification. Importantly it is worth noting that our sample size of 2248 individuals is large enough to ensure a high level of study power (>94%) to detect the small-to-moderate impact of common polymorphisms.

Selection of *RAGE* gene as a CAD-susceptibility candidate is founded on strong biological and genetic bases [3], [17], [20]. The *RAGE* gene is located in the crowded major histocompatibility complex (MHC) class III region, and there is strong evidence supporting a tight linkage between *RAGE* gene rs1800625 and tumor necrosis factor-α gene G-308A polymorphism [21]. Also worth mentioning in the present study is the potential interactions of *RAGE* gene two identified polymorphisms, rs1800625 and rs184003, in susceptibility to CAD. As demonstrated in our single-locus analyses, these two polymorphisms by itself were significantly associated with the risk of developing CAD, especially under the recessive model. Further in haplotype analyses, nearly all haplotypes harboring either risk-conferring allele of two identified polymorphisms had an increased risk for CAD, suggesting the potential existence of locus-to-locus interaction. To shed some light, a promising data-mining analytical approach MDR, which is nonparametric and genetic model-free nature in design [22], was employed, and as expected the aforementioned two polymorphisms constituted the overall best interactive model, reinforcing the results of both single-locus and haplotype analyses. These findings further confirmed our previous claims regarding the informative nature of haplotype approach on the premise of the synergistic effects within polymorphisms [23]. Although residual confounding by incompletely measured or unmeasured physiologic covariates might exist, it seems unlikely that our results could be explained by confounding. In addition, from a biological standpoint, besides the potential impact of promoter rs1800625 on transcriptional regulation [24], it cannot be overlooked that the intronic rs184003 might be functional given the potential regulatory effect of intronic loci on the stability of DNA molecule [25], or alternatively this polymorphism might act as a surrogate marker in linkage disequilibrium with other functional loci in regulatory regions of *RAGE* gene. It is therefore reasonable to hypothesize that the interaction of multiple genetic polymorphisms in *RAGE* gene might play a contributory role in the pathogenesis of CAD in Han Chinese.

Despite the clear strengths of our study, including the relatively large sample size, the angiographically-confirmed CAD patients and controls, and the selection of candidate gene and polymorphisms with strong biological plausibility, the interpretation of our results, however, should be viewed in light of several limitations. First, the retrospective design of this study has inherent drawbacks and precludes causal inferences [26]. Second, we only focused on four common polymorphisms of *RAGE* gene, and it is encouraged to examine more polymorphisms, especially the low-penetrance polymorphisms from other promising CAD-susceptibility genes, such as interleukin-6 gene [27]. More importantly, because CAD is a multifactorial disease, characterizing the interaction of multiple polymorphisms from different chromosomes is deemed as an effective approach to elucidate final genetic architecture of complex disease [28]. Third, the MDR method used in this study has some underling drawbacks including computational intensiveness, indistinct interpretation, lack of sensitivity, and heterogeneity-free assumption [22], [29]. Fourth, we recruited study individuals aged more than 50 years, and future larger association studies in a young population of CAD patients are of specific interest, because genetic factors may have greater contribution to those suffering premature CAD and in the absence of strong environmental risk factors [30]. Last but not the least, the fact that our study population was of Han Chinese descent limited the generalizability of our findings, calling for further confirmation in other ethnic groups.

Taken together, our findings provided strong evidence for the potentially contributory roles of *RAGE* genetic polymorphisms, especially in the context of locus-to-locus interaction, in the pathogenesis of CAD among 2248 northeastern Han Chinese. Moreover, corrections from statistical and practical points of view established the robustness of our findings. For practical reasons, large, well-designed longitudinal studies attempting to account for gene-gene and gene-environment interactions, as well as studies seeking to provide biological or clinical implications, are warranted in the future investigation.

## References

[1] Hegab Z, Gibbons S, Neyses L, Mamas MA (2012) Role of advanced glycation end products in cardiovascular disease. World J Cardiol 4: 90–102. 22558488 10.4330/wjc.v4.i4.90 PMC3342583

[2] Shrikhande GV, Scali ST, da Silva CG, Damrauer SM, Csizmadia E, et al (2010) O-glycosylation regulates ubiquitination and degradation of the anti-inflammatory protein A20 to accelerate atherosclerosis in diabetic ApoE-null mice. PLoS One 5: e14240. 21151899 10.1371/journal.pone.0014240 PMC2997780

[3] Pollreisz A, Hudson BI, Chang JS, Qu W, Cheng B, et al (2010) Receptor for advanced glycation endproducts mediates pro-atherogenic responses to periodontal infection in vascular endothelial cells. Atherosclerosis 212: 451–456. 20701913 10.1016/j.atherosclerosis.2010.07.011 PMC2952730

[4] Ramasamy R, Yan SF, Schmidt AM (2009) RAGE: therapeutic target and biomarker of the inflammatory response – the evidence mounts. J Leukoc Biol 86: 505–512. 19477910 10.1189/jlb.0409230

[5] Volz HC, Laohachewin D, Seidel C, Lasitschka F, Keilbach K, et al (2012) S100A8/A9 aggravates post-ischemic heart failure through activation of RAGE-dependent NF-kappaB signaling. Basic Res Cardiol 107: 250. 22318783 10.1007/s00395-012-0250-z

[6] Neeper M, Schmidt AM, Brett J, Yan SD, Wang F, et al (1992) Cloning and expression of a cell surface receptor for advanced glycosylation end products of proteins. J Biol Chem 267: 14998–15004. 1378843

[7] Falcone C, Emanuele E, D'Angelo A, Buzzi MP, Belvito C, et al (2005) Plasma levels of soluble receptor for advanced glycation end products and coronary artery disease in nondiabetic men. Arterioscler Thromb Vasc Biol 25: 1032–1037. 15731496 10.1161/01.ATV.0000160342.20342.00

[8] Selejan SR, Poss J, Hewera L, Kazakov A, Bohm M, et al (2012) Role of receptor for advanced glycation end products in cardiogenic shock. Crit Care Med 40: 1513–1522. 22430238 10.1097/CCM.0b013e318241e536

[9] Park HJ, Baek JY, Shin WS, Kim DB, Jang SW, et al (2011) Soluble receptor of advanced glycated endproducts is associated with plaque vulnerability in patients with acute myocardial infarction. Circ J 75: 1685–1690. 21576827 10.1253/circj.cj-10-1248

[10] Cai XY, Lu L, Wang YN, Jin C, Zhang RY, et al (2011) Association of increased S100B, S100A6 and S100P in serum levels with acute coronary syndrome and also with the severity of myocardial infarction in cardiac tissue of rat models with ischemia-reperfusion injury. Atherosclerosis 217: 536–542. 21663912 10.1016/j.atherosclerosis.2011.05.023

[11] Gao J, Shao Y, Lai W, Ren H, Xu D (2010) Association of polymorphisms in the RAGE gene with serum CRP levels and coronary artery disease in the Chinese Han population. J Hum Genet 55: 668–675. 20668462 10.1038/jhg.2010.85

[12] Khanna M, Park P, Zirvi M, Cao W, Picon A, et al (1999) Multiplex PCR/LDR for detection of K-ras mutations in primary colon tumors. Oncogene 18: 27–38. 9926917 10.1038/sj.onc.1202291

[13] Stram DO, Leigh Pearce C, Bretsky P, Freedman M, Hirschhorn JN, et al (2003) Modeling and E-M estimation of haplotype-specific relative risks from genotype data for a case-control study of unrelated individuals. Hum Hered 55: 179–190. 14566096 10.1159/000073202

[14] Pattin KA, White BC, Barney N, Gui J, Nelson HH, et al (2009) A computationally efficient hypothesis testing method for epistasis analysis using multifactor dimensionality reduction. Genet Epidemiol 33: 87–94. 18671250 10.1002/gepi.20360 PMC2700860

[15] Hahn LW, Ritchie MD, Moore JH (2003) Multifactor dimensionality reduction software for detecting gene-gene and gene-environment interactions. Bioinformatics 19: 376–382. 12584123 10.1093/bioinformatics/btf869

[16] Torres MC, Beltrame MH, Santos IC, Picheth G, Petzl-Erler ML, et al (2012) Polymorphisms of the promoter and exon 3 of the receptor for advanced glycation end products (RAGE) in Euro- and Afro-Brazilians. Int J Immunogenet 39: 155–160. 22133449 10.1111/j.1744-313X.2011.01073.x

[17] Wang J, Zou L, Song Z, Lang X, Huang S, et al (2012) Meta-analysis of RAGE gene polymorphism and coronary heart disease risk. PLoS One 7: e50790. 23236395 10.1371/journal.pone.0050790 PMC3516500

[18] Evangelou E, Ioannidis JP (2013) Meta-analysis methods for genome-wide association studies and beyond. Nat Rev Genet 14: 379–389. 23657481 10.1038/nrg3472

[19] Pineda-Krch M, Lehtila K (2004) Costs and benefits of genetic heterogeneity within organisms. J Evol Biol 17: 1167–1177. 15525396 10.1111/j.1420-9101.2004.00808.x

[20] Kalea AZ, Schmidt AM, Hudson BI (2009) RAGE: a novel biological and genetic marker for vascular disease. Clin Sci (Lond) 116: 621–637. 19275767 10.1042/CS20080494

[21] Laki J, Kiszel P, Vatay A, Blasko B, Kovacs M, et al (2007) The HLA 8.1 ancestral haplotype is strongly linked to the C allele of −429T>C promoter polymorphism of receptor of the advanced glycation endproduct (RAGE) gene. Haplotype-independent association of the −429C allele with high hemoglobinA1C levels in diabetic patients. Mol Immunol 44: 648–655. 16504296 10.1016/j.molimm.2006.01.011

[22] Gui J, Andrew AS, Andrews P, Nelson HM, Kelsey KT, et al (2010) A simple and computationally efficient sampling approach to covariate adjustment for multifactor dimensionality reduction analysis of epistasis. Hum Hered 70: 219–225. 20924193 10.1159/000319175 PMC2982850

[23] Niu WQ, Zhao HY, Zhou L, Dai XX, Wang DY, et al (2009) Interacting effect of genetic variants of angiotensin II type 1 receptor on susceptibility to essential hypertension in Northern Han Chinese. J Hum Hypertens 23: 68–71. 18633425 10.1038/jhh.2008.77

[24] Hudson BI, Stickland MH, Futers TS, Grant PJ (2001) Effects of novel polymorphisms in the RAGE gene on transcriptional regulation and their association with diabetic retinopathy. Diabetes 50: 1505–1511. 11375354 10.2337/diabetes.50.6.1505

[25] Roy SW, Irimia M (2008) Intron mis-splicing: no alternative? Genome Biol 9: 208. 18304372 10.1186/gb-2008-9-2-208 PMC2374719

[26] Gu M, Dong X, Zhang X, Wang X, Qi Y, et al (2012) Strong association between two polymorphisms on 15q25.1 and lung cancer risk: a meta-analysis. PLoS One 7: e37970. 22701590 10.1371/journal.pone.0037970 PMC3368941

[27] Niu W, Liu Y, Qi Y, Wu Z, Zhu D, et al (2012) Association of interleukin-6 circulating levels with coronary artery disease: a meta-analysis implementing mendelian randomization approach. Int J Cardiol 157: 243–252. 22261689 10.1016/j.ijcard.2011.12.098

[28] Niu W, Qi Y (2012) Matrix metalloproteinase family gene polymorphisms and risk for coronary artery disease: systematic review and meta-analysis. Heart 98: 1483–1491. 22689717 10.1136/heartjnl-2012-302085

[29] Moore JH, Ritchie MD (2004) STUDENTJAMA. The challenges of whole-genome approaches to common diseases. JAMA 291: 1642–1643. 15069055 10.1001/jama.291.13.1642

[30] Zintzaras E, Raman G, Kitsios G, Lau J (2008) Angiotensin-converting enzyme insertion/deletion gene polymorphic variant as a marker of coronary artery disease: a meta-analysis. Arch Intern Med 168: 1077–1089. 18504336 10.1001/archinte.168.10.1077

